# Development of a Novel Blood‐Based Brain‐Derived Tau Biomarker for Alzheimer's Disease and Brain Injury

**DOI:** 10.1002/alz70856_106807

**Published:** 2026-01-08

**Authors:** Wasiu Gbolahan Balogun, Michel N Nafash, Anuradha Sehrawat, Xuemei Zeng, Sarah E. Svirsky, Alexandra Gogola, Julia K. Kofler, Dana L Tudorascu, C. Elizabeth Shaaban, Jennifer H Lingler, Tharick A Pascoal, William E Klunk, Victor L. Villemagne, Sarah B Berman, Beth E. Snitz, Milos D. Ikonomovic, Ann D Cohen, M. Ilyas Kamboh, Ava M Puccio, Oscar L Lopez, Thomas K Karikari

**Affiliations:** ^1^ University of Pittsburgh, Pittsburgh, PA, USA; ^2^ University of Pittsburgh School of Medicine, Pittsburgh, PA, USA; ^3^ University of Pittsburgh, School of Medicine, Pittsburgh, PA, USA

## Abstract

**Background:**

Brain‐derived tau (BD‐tau) is an emerging blood‐based biomarker for neurodegeneration associated with Alzheimer's disease (AD). Currently, there are limited BD‐tau assays available for research and clinical use. The only existing commercial assay is still undergoing validation, limiting its acceptance among researchers. To improve access to this crucial biomarker for AD, our laboratory at the University of Pittsburgh has developed a novel blood‐based immunoassay for BD‐tau, referred to as Pitt‐BD‐tau. Following comprehensive analytical validation, we evaluated the clinical utility of the assay in two independent cohorts.

**Method:**

We established a three‐step ultra‐sensitive BD‐tau immunoassay using the Quanterix HDX Single Molecule Array (Simoa) platform. The analytical validation examined dilution linearity, specificity, precision, detection limits, and spike recovery. Clinical validation was conducted in two cohorts: one from the memory clinic at the University of Pittsburgh Alzheimer's Disease Research Center (Pitt‐ADRC) and another from the Pittsburgh traumatic brain injuries (TBI cohort). We compared the performance of the Pitt‐BD‐tau assay with that of the commercially available Quanterix BD‐tau assay. Spearman correlation was used to establish relationships between the continuous variables.

**Results:**

The newly developed Pitt‐BD‐tau assay demonstrated high validation metrics, achieving between‐run stability of 95% when aliquots from four independent plasma samples were analyzed across five analytical runs. It also exhibited strong dilution linearity when diluted 4‐fold and achieved over 90% recovery when spiked. Robust correlations between the Pitt‐BD‐tau and Quanterix BD‐tau assays were observed in both cohorts, with correlation coefficients of 0.85 and 0.97 for Pitt‐ADRC and TBI cohort, respectively. Additionally, in the TBI cohort, the assay differentiated between control, chronic‐mixed TBI, and acute‐severe TBI individuals indicating a significant association with TBI with (*p* <0.0001) similar to the commercial assay.

**Conclusion:**

Our findings highlight the significant potential of the Pitt‐BD‐tau assay, the first developed by an academic laboratory. This assay demonstrates reliability, reproducibility, and the ability to identify individuals with TBI. However, more still need to be done for using the assay to identify individuals with biological evidence of Alzheimer's disease.

